# Local variation in brain temperature explains gender-specificity of working memory performance

**DOI:** 10.3389/fnhum.2024.1398034

**Published:** 2024-07-26

**Authors:** Jacek Rogala, Joanna Dreszer, Marcin Sińczuk, Łukasz Miciuk, Ewa Piątkowska-Janko, Piotr Bogorodzki, Tomasz Wolak, Andrzej Wróbel, Marek Konarzewski

**Affiliations:** ^1^Centre for Research on Culture, Language, and Mind, University of Warsaw, Warsaw, Poland; ^2^The Centre for Systemic Risk Analysis, University of Warsaw, Warsaw, Poland; ^3^Faculty of Philosophy and Social Sciences, Institute of Psychology, Nicolaus Copernicus University in Toruń, Toruń, Poland; ^4^Nalecz Institute of Biocybernetics and Biomedical Engineering, Polish Academy of Sciences, Warsaw, Poland; ^5^Bioimaging Research Center, World Hearing Center, Institute of Physiology and Pathology of Hearing, Kajetany, Poland; ^6^Nencki Institute of Experimental Biology, Warsaw, Poland; ^7^Faculty of Philosophy, University of Warsaw, Warsaw, Poland; ^8^Faculty of Biology, University of Białystok, Białystok, Poland

**Keywords:** biological sciences, neuroscience, brain temperature, working memory, gender differences, magnetic resonance spectroscopic thermometry, functional magnetic resonance imaging

## Abstract

**Introduction:**

Exploring gender differences in cognitive abilities offers vital insights into human brain functioning.

**Methods:**

Our study utilized advanced techniques like magnetic resonance thermometry, standard working memory n-back tasks, and functional MRI to investigate if gender-based variations in brain temperature correlate with distinct neuronal responses and working memory capabilities.

**Results:**

We observed a significant decrease in average brain temperature in males during working memory tasks, a phenomenon not seen in females. Although changes in female brain temperature were significantly lower than in males, we found an inverse relationship between the absolute temperature change (ATC) and cognitive performance, alongside a correlation with blood oxygen level dependent (BOLD) signal change induced by neural activity. This suggests that in females, ATC is a crucial determinant for the link between cognitive performance and BOLD responses, a linkage not evident in males. However, we also observed additional female specific BOLD responses aligned with comparable task performance to that of males.

**Discussion:**

Our results suggest that females compensate for their brain’s heightened temperature sensitivity by activating additional neuronal networks to support working memory. This study not only underscores the complexity of gender differences in cognitive processing but also opens new avenues for understanding how temperature fluctuations influence brain functionality.

## Introduction

The cognitive abilities of men and women have been under scientific scrutiny for over a century, with a key area of interest being working memory. Playing a crucial role in brain information transfer, working memory affects other cognitive domains, implying that a gender difference in working memory could have broader impacts on cognitive functioning. Yet, behavioral investigations into the influences of gender have yielded inconsistent results. Some meta-analyses suggest a female advantage in verbal working memory (Asperholm et al., 2019; Hirnstein et al., 2023), while others demonstrate no performance differences (Voyer et al., 2017), a female disadvantage (Zilles et al., 2016), or show mixed results pointing to an advantage in males or females depending on the working memory task (Voyer et al., 2021).

Sexual dimorphism in working memory performance appears to be functionally linked to underlying physiology. Previous research has identified sex-based differences in cerebral blood flow (Rodriguez et al., 1988; Daniel et al., 1989; Esposito et al., 1996; Aanerud et al., 2017) and synaptic strength (Aanerud et al., 2017). Remarkably, even when no performance differences between genders are reported, distinct brain activation patterns persist in males and females (Alarcón et al., 2014). A plausible explanation is the variation of brain temperature resulting from obligatory heat cogeneration by metabolic processes underlying neuronal activation. Given that temperature impacts all physical and biochemical processes at the rate of the Van’t Hoff coefficient (McCullough et al., 1999), even small variations, less than one degree Celsius, might adversely affect neuronal activity (Kim et al., 2022). Importantly, the observed daily variations in human brain temperature often exceeded four degrees Celsius (Rzechorzek et al., 2022), and, therefore, may significantly affect neuronal cognitive processes.

Interestingly, the main factors driving changes in brain temperature are neuronal activity and the cooling effect of incoming blood (Kiyatkin et al., 2002). It’s therefore plausible that changes in brain temperature could differ across genders and differentially affect male and female cognitive performance and overall brain activity. However, research into this specific area is still in its infancy, largely because investigations involving human brain temperature typically require invasive techniques. These procedures present ethical considerations and potential risks to patients, such as damage to nervous tissues, making them unsuitable for studies exploring brain activity or cognitive performance.

In theory, one could estimate brain temperature’s effects on cognitive performance using core body temperature as a surrogate. However, the correlation between core body and brain temperature in humans is modest (Thrippleton et al., 2014). Therefore, attempting to infer brain temperatures from core body temperatures could lead to considerable inaccuracies. A more appropriate approach for healthy individuals is to employ non-invasive techniques accurate in examining three-dimensional objects with high water content, such as biological tissues. Magnetic resonance spectroscopy thermometry (MRS-t) is one such technique, which uses magnetic resonance imaging (MRI) scanners to measure temperature-dependent nuclear magnetic resonance (NMR) parameters. The same MRI scanners used for temperature estimation can be used for functional Magnetic Resonance Imagining (fMRI) brain imaging with the use of the blood oxygen level dependent (BOLD) signal. The BOLD signal reflects regional differences in cerebral blood flow induced by regional brain activity, thereby providing essential information for studying human brain functions. Simultaneous fMRI and electrophysiological recordings have validated that BOLD changes reflect aspects of neural responses elicited by a stimulus in relevant brain areas (Logothetis and Wandell, 2004). The MRS-t and fMRI can be smoothly integrated within a single fMRI session to decode the interplay between gender, brain temperature variations, and brain activity. Continuous advancements over the past three decades have increased the accuracy and reliability of MRS-t, as confirmed by numerous studies (Winter et al., 2016; Lutz and Bernard, 2020; Zhang et al., 2020; Sung et al., 2022).

In our study, we investigated whether gender-specific variations in brain temperatures induced by a working memory task could explain the different neuronal responses in men and women regardless of observed differences in the performance. More specifically, we predicted that task-induced variations in brain temperature may drive gender-specific rates of metabolic processes in activated brain regions possibly affecting cognitive performance. To explore these associations, we combined n-back tasks (a widely used and thoroughly studied experimental cognitive paradigm; Gevins and Cutillo, 1993; Finc et al., 2017) with MRS-t to estimate variations in brain temperature, and used fMRI to record activity in 16 brain regions of interest (ROIs) involved with the task during a single MRI session.

## Materials and methods

### Participants

Sample size was estimated with G*Power 3.1.9.7 for power of 0.90 and an effect size of f2 = 0.50 for the following analyses: two-way mixed ANOVA, moderation analysis and MANOVA. G*Power estimation indicated sample sizes of *N* = 34, *N* = 61, *N* = 64 participants, respectively. Details of the calculations are presented. Accounting for possible exclusion of participants due to artifacts and/or cancellation of participation in the study we recruited 65 participants. Finally 63 right-handed healthy adults (32 females) took part in this study with a mean age of 28 years (SD = 5.5 years, range = 21–42 years). Participants were paid for their time. They were homogeneous in terms of their socioeconomic status and education level, and the majority were students. Following the ethical principles for medical research involving human subjects, ethical approval was granted by the local ethics committee at the Nicolaus Copernicus University in Torun (approval no 3/2018, dated 6.04.2018) and all participants provided informed consent after a thorough explanation of the study’s procedures and risks. Participants were included in this experiment after verifying the absence of contraindications to MRI examinations (e.g., claustrophobia, cochlear implants, metal fragments in the participants’ eye or head, pacemakers) as well as other conditions posing a potential risk for the participants.

### Acquisition of MRI data

The MRI data acquisition was carried out using a 3-T GE Discovery 750w scanner (CNS Lab IBBE PAS) fitted with a standard receiver, 8-channel head coil. Room temperature (20.5°C, standard deviation [SD] ± 0.5°C) and lighting were constant. After standard shimming, a high-resolution structural MRI sequence (TR/TE = 6.936 ms/2.968 ms, matrix 256 × 256, voxel 1.0547 × 1.0547 × 1.2 mm3) was acquired for improved voxel localization. For MRS-t measurements a single-voxel Point Resolved Spectroscopy (PRESS) sequence was performed on a voxel of interest (VOI) (20 × 20 × 20 mm^3^) positioned in the right inferior parietal lobule (rIPL). This large VOI ensures a reasonable signal-to-noise ratio to gain sufficient signal for further data processing. It is also one of the regions of interest (ROIs) identified by Owen et al. (2005) in their meta-analysis devoted to n-back working memory tasks. We used single voxel spectroscopy (SVS) for its high signal-to-noise ratio during a relatively short scan time, and the compact imaging area allowed shimming with high-quality spectra suitable for peak parameterization allowing higher precision of temperature estimation than spectroscopy imaging methods (Bertoldo et al., 2013). The scan parameters for the PRESS were as follows: TR = 1,500 ms, TE = 30 ms, bandwidth 5 kHz, 4,096 points, NEX = 8, with 96 averages and a scan time of 3 min. For more precise data analysis, water suppression during the scanning sequence was applied. After MRS-t scanning, three fMRI sessions were collected consisting of 480 t2*-weighted gradient echo planar imaging sequence scans (TR = 2,500 ms, TE = 25 ms, voxel 3 × 3 × 3 mm^3^). During the three sessions, lasting 4 min and 25 s each, participants performed n-back tasks (Finc et al., 2017). After fMRI sessions, a second pair of PRESS scans were conducted using the same parameters. The total time scheduled for MRI acquisitions was approximately 60 min for each participant: 15 min for participant preparation and 45 min for MRI acquisition consisting of structural scan, MRS-t scan, three fMRI sessions and another MRS-t scan. In total we acquired 128 MRS-t measurements (2 for each of 64 participants).

### *In vivo* temperature measurements

**Figure 1:**
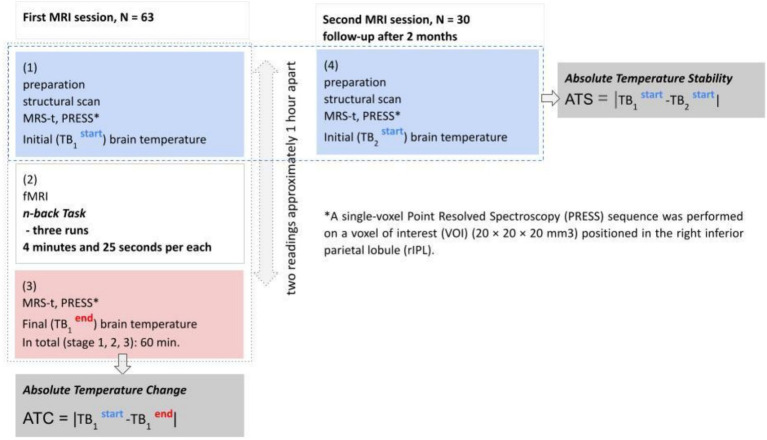
Experimental schema.

There were two sessions of *in vivo* brain temperature measurements planned. First session included: initial temperature measurement (TB_1_^start^), functional scan during working memory task and final temperature measurement (TB_1_^end^). Second session (follow-up after 2 months) was planned for assessment of stability of brain temperature (ATS) and included only initial brain temperature measurement (TB_2_^start^). The second session attended only 30 participants (18 men). The both MRI sessions were scheduled between 8:00 am and 4:00. In the first session the majority of participants (48 out of 63 finally participating in the study) were scanned between 9:30 and 1:30 pm, while in the second session the majority (20 out of 30) were scanned between 10 am and 1 pm. The differences in initial temperature measured during the first (TB_1_^start^) and second (TB_2_^start^) sessions were also used to assess the effect of daytime of brain temperature measurements on its stability (ATS = |TB_1_^**start**^ -TB_2_^**start**^|). The experiment schema is shown on Figure 1.

All brain temperature readings in the first session were taken just before and after the memory task. This choice was justified by the following factors: first, the task condition induces both: increased neuronal activity leading to an increase in temperature, and an influx of cooler blood causing a decrease in temperature above the initial value due to excessive blood influx. The response of this dynamic system to the additional heat induced by RF pulses used in MRI and MRS (Formica and Silvestri, 2004; van der Graaf, 2010) is unknown. Changes of the blood flow in response to the two heat waves and their effects on changes of brain temperature, neuronal activity and memory performance have never been studied and are difficult to assess. Second, prolonged task duration can affect both neural and behavioral activity, making it difficult to separate. Taking temperature readings just before and just after the task frees the measurements from the influence of these additional uncontrolled interference factors.

To assess and account for potential brain body temperature correlations we also took body temperature of the participants. Body temperature was recorded three times before the examination using an infrared ear thermometer (Braun ThermoScan® 3) in the right ear. The participants were fitted comfortably inside the scanner and equipped with earplugs, their head was positioned centrally within the coil, and supported by padding and foam to ensure minimal movement in any plane. Subsequently, the acquisition of the data was performed (see Acquisition of MRI data section for detailed description). On completion of the scan, three additional temperature measurements in the right ear were obtained.

### MRS-t data preprocessing and analysis

The preprocessing and temperature estimations were performed using house-developed automated software based on FID-A (Simpson et al., 2017), an open-source software package written under a Matlab™ platform (MathWorks, Natick, MA, USA). After the preprocessing of the MRS-t spectra (Near et al., 2021), covering the following steps: Fourier transformation, RF coil combination, signal averaging, and phase correction. Individual metabolites and water peaks were parameterized using Lorentzian fitting. From these parameterizations, we obtained the chemical shifts of the metabolites and water peaks. These chemical shifts were then utilized in temperature estimations according to the following equation: T = 277.2–90.1*(δWater - δNAA).

where:

T - is the temperature (°C),

δWater - is the chemical shift of the partially suppressed water (H20) peak, and

δNAA - is the chemical shift of the N-acetylaspartate (NAA) peak.

The constant coefficients in the equation were measured with a temperature-controlled phantom (Sińczuk et al., 2023) containing: N-acetylaspartate, choline, and creatine metabolites in the proportions as observed in the healthy human brain. In light of recent research (Dong et al., 2022), which highlighted the potential for systematic errors in repeated measurements, we collected data on the chemical shift of water and NAA peaks from the same spectra. The accuracy of temperature estimations, according to past research is within approximately 0.5°C (Verius et al., 2019).

Due to a short period of temperature monitoring (two readings 1 h apart during the first session, Figure 1), the regular cosinor analysis required to identify circadian temperature rhythm and amplitude had to be adjusted (Lu et al., 2021). We used absolute temperature differences as the proxy for amplitude.

### Absolute temperature change (ATC)

Because each cellular and molecular process is likely to have a different temperature dependence, defined as the rate ratio of a given process taking place at temperatures differing by 10 units (Q10, Mundim et al., 2020), predicting neuronal activity with temperature changes is not trivial. Available reports on temperature-dependent neuronal activity show a plethora of possible responses, for instance, Masino and Dunwiddie (1999) found that increasing the temperature markedly inhibits excitatory synaptic transmissions, while other investigations showed that lower temperatures caused slower nerve conduction velocities and increased nerve potential amplitudes (Denys, 1991). Yet, another study indicated that field excitatory potentials were linearly related to brain temperature (Moser et al., 1993), while Boulant and Hardy (1974) found both negative and positive coefficients for temperatures and firing rates. Thus, to account for differentiated neuronal responses during temperature changes, we adopted ATC as the absolute value of the difference between initial and final brain temperatures (ATC = |TB_1_^**start**^ -TB_1_^**end**^|). Similar measures - of brain temperature amplitudes – are commonly used in longitudinal brain temperature monitoring in clinical and research practice (Kiyatkin et al., 2002; Kiyatkin, 2019; Rzechorzek et al., 2022).

### fMRI (blood oxygen level dependent) data preprocessing and analysis

The data preprocessing pipeline was done using the SPM12 toolbox (Wellcome Department of Imaging Neuroscience, Institute of Neurology, London, UK) running on Matlab (R2016a) (Mathworks, Natick, MA, USA). Functional images were corrected for acquisition time and spatially realigned to the mean image to correct for interscan head motion. The structural T1-weighted image was co-registered with the mean functional image. Functional, gray matter, white matter, and CSF images were spatially normalized to the MNI template using new unified normalization segmentation. One participant and five single functional scans from other participants were excluded from further analysis due to excessive head motion artifacts (more than 10 in outlier scans), thus, the final sample consisted of 63 subjects. No significant differences in the number of outlier scans were found between 1-back and 2-back tasks [*t*(63) = 0.38, *p* = 0.82].

The I-level analysis of the blood oxygen level dependent (BOLD) signals was done under an SPM12 general linear model (GLM) framework. A design matrix consisted of three task-related regressors corresponding to 0-back, 1-back, and 2-back scanning sessions, session-specific effects, and nuisance variables like subject head movement. A linear combination of effect sizes for task regressors (1- vs. 0-back and 2- vs. 1-back) was chosen with contrast vectors for further group-level analysis. On II-level analysis, additional regressors such as initial brain temperature, final brain temperature, change in temperature during the study, and gender were defined. Based on previous publications (Owen et al., 2005), 16 ROIs were selected for which the mean activity values were determined for the two mentioned BOLD contrasts. ROIs identified by Owen et al. (2005) devoted to the verbal variant of n-back working memory tasks included the bilateral premotor cortex, left dorsal anterior cingulate cortex, right dorsolateral prefrontal cortex, left ventrolateral prefrontal cortex (frontal inferior operculum and rolandic operculum), left and right frontal poles, right medial posterior parietal cortex, left and right IPL (inferior parietal and anterior cingulum), and three sites in the medial and lateral cerebellum and right thalamus.

### Working memory task

To assess working memory performance, we used classical n-back tasks. Details of the task used in the current study were described in our previous work (Finc et al., 2017). In short, a visual letter-based n-back task (Gevins and Cutillo, 1993) requires participants to continuously monitor, update, and manipulate information held in memory (Owen et al., 2005). In our study, the stimulus consisted of the first five letters of the Latin alphabet (A–E, Kearney-Ramos et al., 2014), presented in pseudorandom order in the 0-, 1-, and 2-back conditions. The presentation of each letter (500 ms) was preceded by a fixation cross (1,500 ms) presented in the center of the screen. Each execution (RUN) of the task consisted of 10 blocks (30 s per block, 12 trials with 25% targets), in which 0-, 1-, and 2-back conditions alternated. Before each block, brief instructions (2 s) were displayed to inform the participant about the current task. The task consisted of three RUNs with a total length of 12 min; 480 functional epi scans were performed in total. Depending on the conditions, participants were asked to indicate whether the currently presented letter was the same or different from the letter presented one or two trials earlier. Participants had 2000 ms for each response. The execution and control of the experimental protocol (stimulus delivery and response recording) were performed by Presentation® software (Neurobehavioral Systems, Albany, NY), version 17.2. Visual stimuli were displayed on a monitor and presented to the participant in a mirror. Participants were instructed to respond as quickly and accurately as possible by pressing one of two buttons with the thumb of their right hand (target letter - right button, non-target letter - left button).

### Behavioral performance evaluation

To evaluate a participant’s performance in the n-back tasks, we used the sensitivity index d prime (Macmillan and Creelman, 1990) averaged of all RUNs. The d prime stands for the difference between Z transforms of the hit rate and false alarms. The hit rate is defined as the proportion of hits when a signal is present to all target stimuli, and false alarms represent the proportion of responses when a signal is absent to all non-target stimuli. Final scores were adjusted according to the following formulas:

1–1/(2n) for perfect hits and 1/(2n) for zero false alarms, where n was the number of total hits or false alarms (Macmillan and Creelman, 1990).

### Statistical methods

Prior to any analyses all data were tested for normality using the Shapiro–Wilk test, kurtosis, and skewness. Data were also tested for the presence of outliers. The values deviating from the mean by more than three standard deviations were removed.

Comparison of men’s and women’s (between factor) brain temperature measured during the first MRI session (within: TB_1_^start^ and final TB_1_^end^) was performed using two-way mixed ANOVA. Next, to consider the bi-directionality effect of temperature, we used the absolute difference between the initial and final brain temperature changes (ATC). A comparison of the ATC of both groups (females vs. males) was conducted using two independent sample *t*-tests. Gender-related differences in Absolute Temperature Stability (ATS) index were assessed using two independent sample *t*-tests. We also checked gender related differences in the behavioral outcome of the working memory test (average d prime index) using two independent sample t-tests.

Afterward, to verify the first main hypothesis whether there is a interaction effect of gender and ATC on memory performance (average d prime index), we applied a series of moderation analyses for 2-back testing conditions (Model 1, Hayes, 2022, via SPSS, macro PROCESS v. 3.2).

The neural correlates of ATC in task-related ROIs (the second hypothesis), were computed using a series of linear regressions. Results were corrected for false positives using the False Discovery Rate adjustment (Benjamini and Hochberg, 1995) where appropriate unless otherwise stated. Analyses and figures were created using MATLAB 2016a.

Additionally, in order to assess the differences between males and females in Percent Signal Change (PSC) for 16 ROIs (as response variables), MANOVA was applied. The two-way mixed ANOVA, t-tests and MANOVA were conducted using IBM SPSS Statistics Version 29.0.2.0.

## Results

### Brain temperature and absolute temperature difference

**Figure 2:**
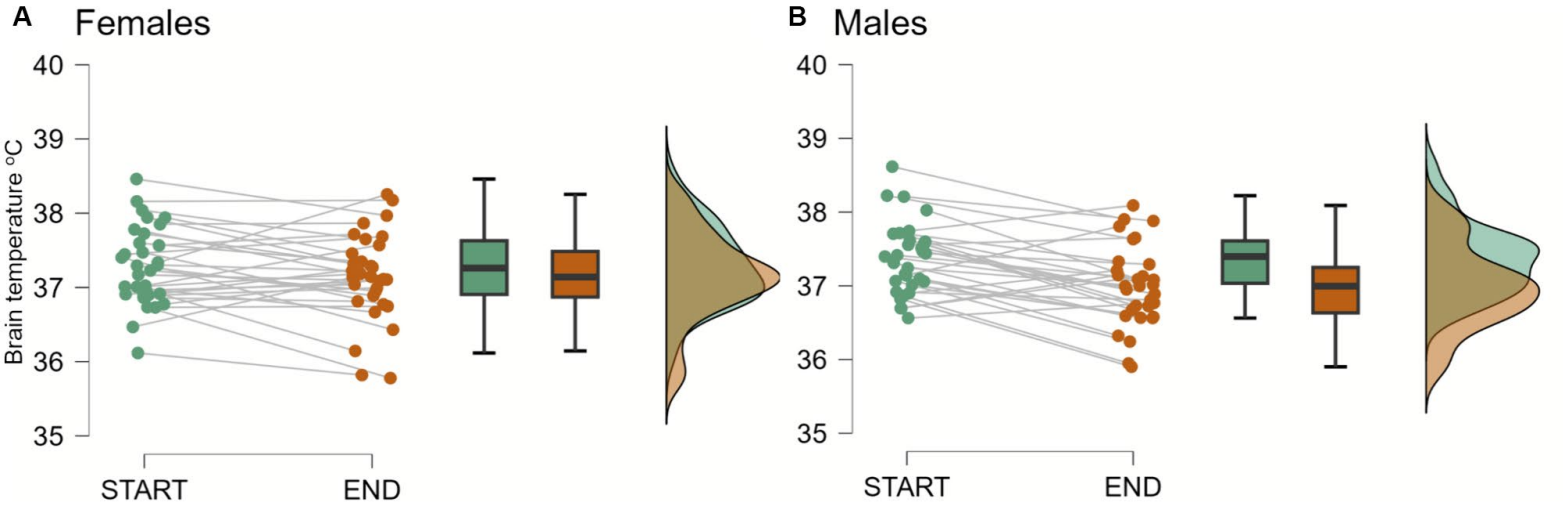
Brain temperature in females and males at the beginning (start) and at the end (end) of a first MRI session. The two-way mixed ANOVA and subsequent *post hoc* tests showed significant decreases in brain temperatures in men (*n* = 31; TB_1_^start^: *M* = 37.37, SD = 0.49, TB_1_^end^: *M* = 36.98, SD = 0.55; *p* < 0.001), but not in women (*n* = 32; TB_1_^start^: *M* = 37.28, SD = 0.53, TB_1_^end^: *M* = 37.14, SD = 0.59; *p* > 0.1).

Initial (TB_1_^start^) and final (TB_1_^end^) brain temperatures, measured in the right parietal lobe, obtained from the first MRI session averaged 37.32°C [*n* = 64 (63); SD = 0.50; min/max: 36.12, 38.62] and 37.07°C (SD = 0.57, min/max: 35.78, 38.25), respectively. TB_1_^end^ was significantly lower than the TB_1_^start^ [*F*(1,61) = 18.70, *p* = <0.001, η_p_^2^ = 0.244]. The two-way mixed ANOVA for the difference between TB_1_^start^ and TB_1_^end^ yielded significant brain temperature * sex interaction [*F*(1,61) = 4.37, *p* = 0.041, η_p_^2^ = 0.067]. Subsequent *post hoc* tests showed a significant decrease in brain temperatures in men (*n* = 31; TB_1_^start^: *M* = 37.37, SD = 0.49, TB_1_^end^: *M* = 36.98, SD = 0.55), but not in women (*n* = 32; TB_1_^start^: *M* = 37.28, SD = 0.53, TB_1_^end^: *M* = 37.14, SD = 0.59, Figure 2).

Both increases and decreases in temperature can affect neuronal activity. To take into consideration this bi-directionality effect of temperatures, we used the absolute difference between the initial and final brain temperature changes (ATC). This approach, detailed in the materials and methods section, will be consistently applied throughout this paper.

**Figure 3:**
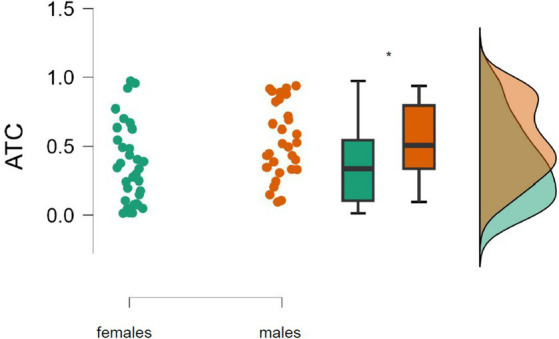
Absolute brain temperature change (ATC) in the right inferior posterior lobule among males (*n* = 31) and females (*n* = 32) during the first MRI session. *Significant difference (*p* = 0.02).

The mean of the ATC of the right inferior posterior lobule was significantly higher in males (0.54°C) than in females (0.38°C), *t* = −2.340, *p* = 0.020, Cohen’s *d* = −0.604 (Figure 3).

### Stability of brain temperature

Comparison of the initial brain temperatures measured during first (TB_1_^start^) and second (TB_2_^start^) MRI session showed no differences neither for the whole group [*t*(29) = 1.04, *p* = 0.308] nor for males [*t*(17) = 0.831, *p* = 0.417] or females [*t*(11) = 0.614, *p* = 0.552]. These results suggest that TB_1_^start^ is stable over time and not susceptible to the time of measurement.

The ATS (Absolute Temperature Stability) calculated as the absolute value of the TB_1_^start^ and TB_2_^start^ difference was 0.5 degrees in both men and women and was not significantly different [*t*(28) = −1.35, *p* = 0.188] suggesting that the difference between the ATCs of men and women was due to differences in their neural activity.

### Distinct effects of brain temperature on gender performance on n-back tasks

**Figure 4:**
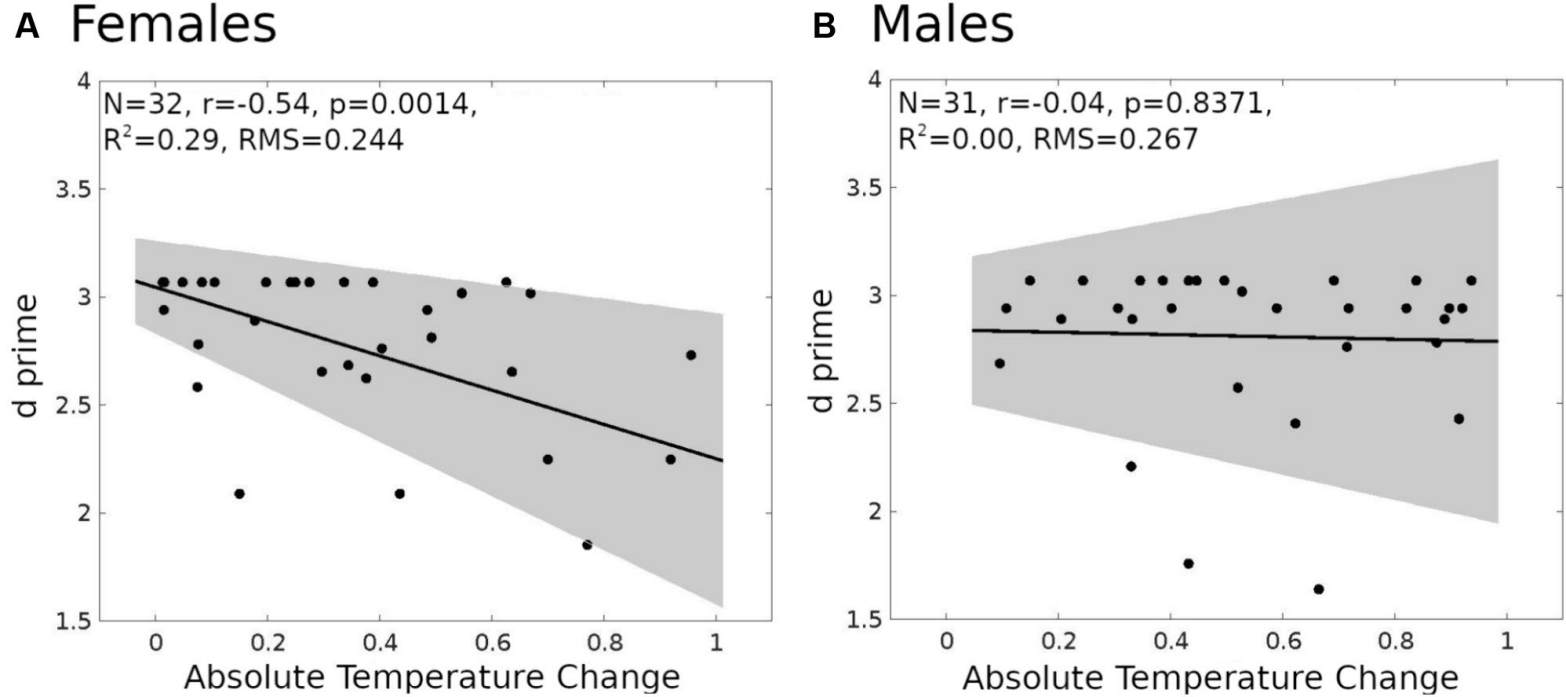
Linear regression models for d prime and absolute temperature change (ATC). **(A)** Female (*n* = 32). **(B)** Male (*n* = 31). The shaded gray region is the 95% confidence level of the regression line.

To explore the potential interactions between gender and ATC on memory performance, we conducted a series of hierarchical regression analyses. The interaction (model 1, PROCESS macro, Hayes, 2022; see the Materials and methods for details) between gender and ATC on predicting working memory performance (measured by d prime index, for details please refer to the behavioral performance evaluation in the materials and methods section) was significant only in the most challenging 2-back testing conditions [*F*(3,59) = 4.14, *p* = 0.01, *R*^2^ = 0.17, *B* = 0.743, SE = 0.348, 95%, CI = [0.05, 1.43], *t* = 2.15, *p* = 0.04]. Interestingly, the association of ATC and d prime was negative in women (simple effect test, slope = −0.7936, SE = 0.2322, 95%, CI = [−1.25, −0.32], *t* = −3.45, *p* = 0.0011, Figure 4A), while in men the association was insignificant (simple effect test, slope = −0.054, SE = 0.25, 95%, CI = [−0.56, 0.46], *t* = −0.2122, *p* = 0.843, Figure 4B).

In the less demanding 1-back task, variations had no effect on ATC and working memory performance in males, females, and the whole group. Most surprisingly, however, despite gender-dependent differences in ATC and ATC-dependent task performance, the behavioral outcomes (d prime, average from three runs) of both genders were the same [*t*(61) = −0.652, *p* = 0.517, in males: *M* = 2.81, SD = 2.37; in females: *M* = 2.75, SD = 0.42]. The most plausible explanation for this could be gender- and ATC-dependent differences in brain activity. To test this hypothesis, we started with analyses of brain activity in task-related cerebral ROIs.

### Neural correlates of ATC in task-related ROIs

**Figure 5:**
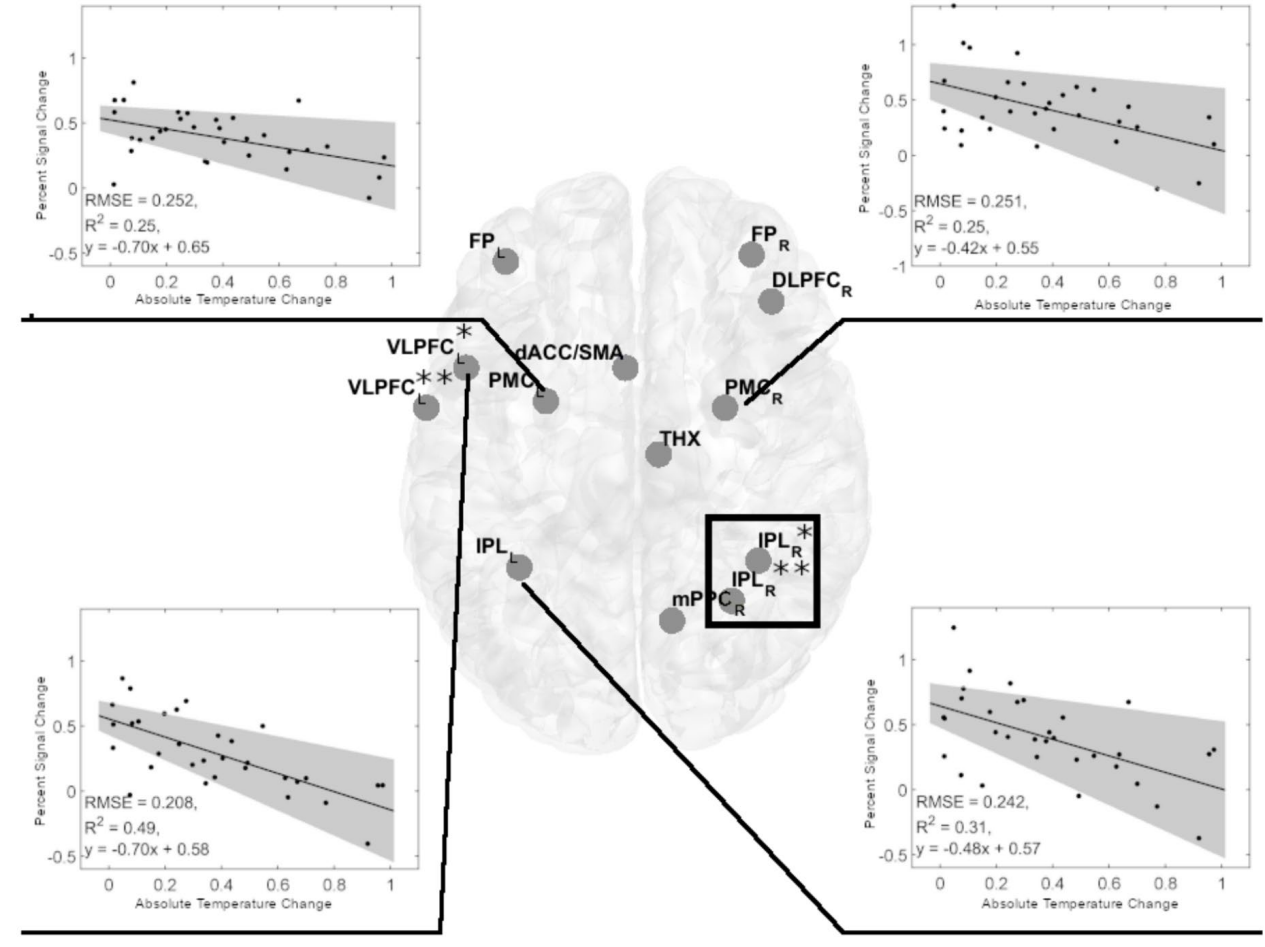
Significant negative linear regression models of Percent Signal Change in cerebral regions of interest (ROIs) for the visual-verbal 2-back tasks and absolute brain temperature changes in females. Linear regression analyses showed four significant models in the 2-back vs. 1-back comparison (indicated by black lines). Inserts show the significant linear regression models for PSC and ACT. All models were significant at *p* < 0.05, FDR corrected. The shaded gray region is the 95% confidence level of the regression line. At VLPC: *denotes frontal inferior operculum, and **denotes Rolandic operculum. At IPL: *denotes inferior parietal gyrus, and **denotes anterior cingulum. The black rectangle denotes the spectroscopy voxel of interest where brain temperature was estimated. FP_L,R 0_, frontal poles; VLPFC_L_, left ventrolateral prefrontal cortex (frontal inferior operculum and Rolandic operculum); dACC_L_, left dorsal anterior cingulate cortex; DLPFC_R_, right dorsolateral prefrontal cortex; PMC_L,R_, bilateral premotor cortex; THX_R_, right thalamus; IPL_L_, left inferior parietal lobule; IPL_R_, two sites in the right inferior parietal lobe; mPPC_R_, the medial posterior parietal cortex.

In women, the observed negative relationship (series of linear regressions, with FDR correction) between ATC and performance in most cognitively demanding 2-back task variants suggested a potentially similar association between ATC and BOLD signal amplitude. Therefore, we analyzed the relationship between ATC and Percent Signal Change (PSC) in 1-back and 2-back task variants for visual-verbal stimuli in the ROIs (Figure 4). These analyses (FDR corrected) yielded significant results only in the more demanding 2-back task variants. We found a negative relationship between ATC and PSCin the left ventrolateral prefrontal cortex (left VLPC, adjusted R^2^ = 0.4696, *p* = 0.0004), the left premotor cortex (left PMC, adjusted R^2^ = 0.2295, *p* = 0.0289), the right premotor cortex (right PMC, adjusted *R*^2^ = 0.2227, *p* = 0.0289), and the left inferior parietal lobule (left IPL, adjusted *R*^2^ = 0.2856, *p* = 0.0231; Figure 5). Identical analyses conducted for males did not yield significant results.

**Figure 6:**
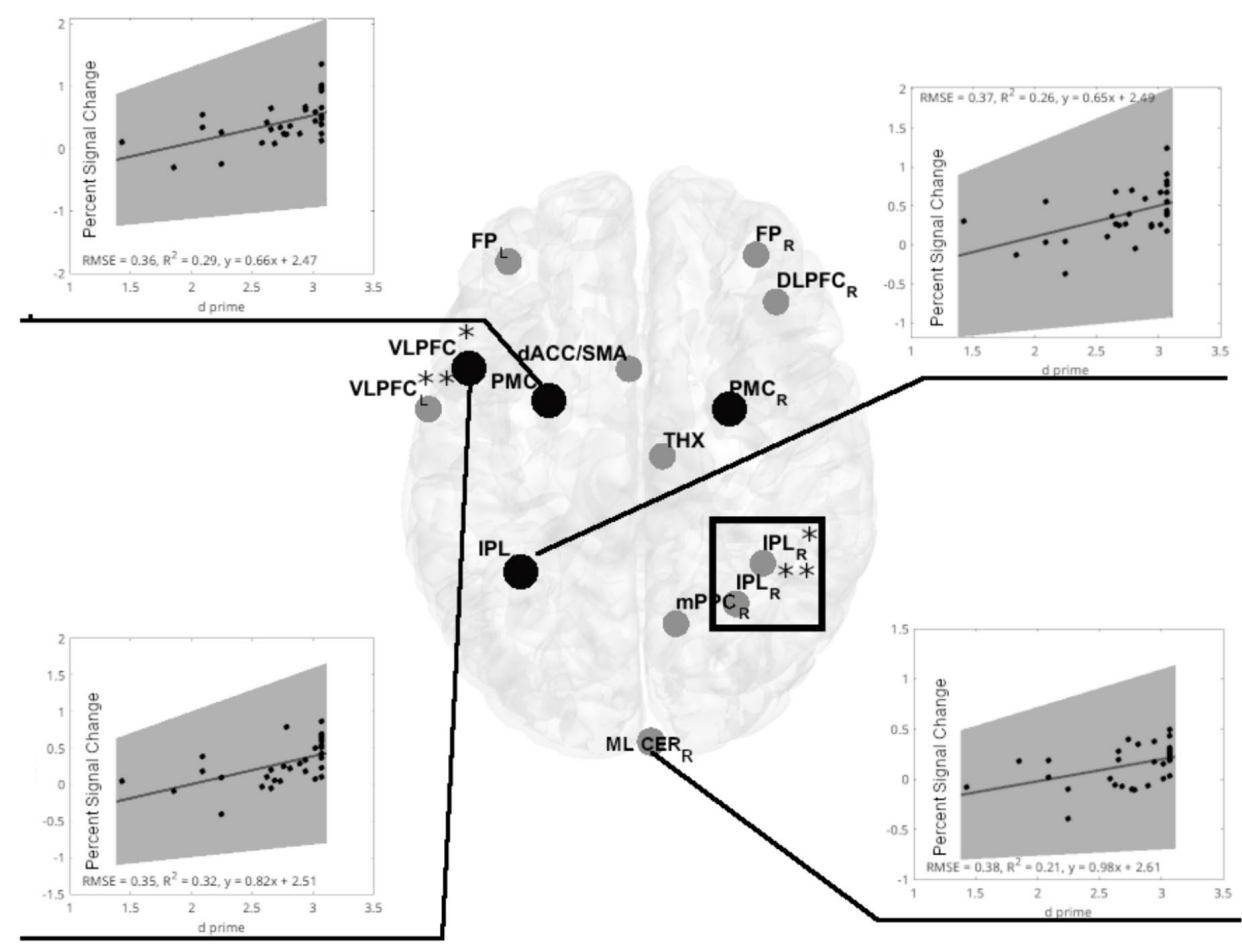
Significant positive linear regression models of d prime vs. PSC in cerebral regions of interest (ROIs) for the visual-verbal 2-back task in females. Linear regression analyses show four significant models in the 2-back vs. 1-back comparisons (indicated by black lines). Out of four significant models, three are located in the same ROIs, identifying an inverse PSC-ATC relationship (marked with black-filled circles). Inserts show significant linear regression models for PSC and d prime. All models were significant at p < 0.05, FDR corrected. The shaded gray region is the 95% confidence level of the regression line. At VLPC: *denotes frontal inferior operculum, and **denotes Rolandic operculum. At IPL: *denotes inferior parietal gyrus, and **denotes anterior cingulum. The black rectangle denotes the spectroscopy voxel of interest where brain temperature was estimated. FP_L,R 0_, frontal poles; VLPFC_L_, left ventrolateral prefrontal cortex (frontal inferior operculum and Rolandic operculum); dACC_L_, left dorsal anterior cingulate cortex; DLPFC_R_, right dorsolateral prefrontal cortex; PMC_L,R_, bilateral premotor cortex; THX_R_, right thalamus; IPL_L_, left inferior parietal lobule; IPL_R_, two sites in the right inferior parietal lobe; mPPC_R_, the medial posterior parietal cortex; ML CER_R_, R-Medial and lateral cerebellum.

An inverse relationship between d prime and ATC (Figure 4), along with an inverse relationship between PSC and ATC (Figure 4), suggests that both relationships lead to a positive relationship between d prime and PSC, driven by ATC. Indeed, in women, we observed a positive PSC-d prime relationship in following ROIs: left VLPFC (adjusted *R*^2^ = 0.285, *p* = 0.012, FDR corrected), left PMC (adjusted *R*^2^ = 0.273, p = 0.012, FDR corrected), and left IPL (adjusted *R*^2^ = 0.228, *p* = 0.0203, FDR corrected, Figure 5). The fourth ROI was located in the right medial and lateral cerebellum (adjusted *R*^2^ = 0.176, *p* = 0.0432, FDR corrected, Figure 6). Conversely, correlation analyses performed separately for men found none of the correlations to be significant.

**Figure 7:**
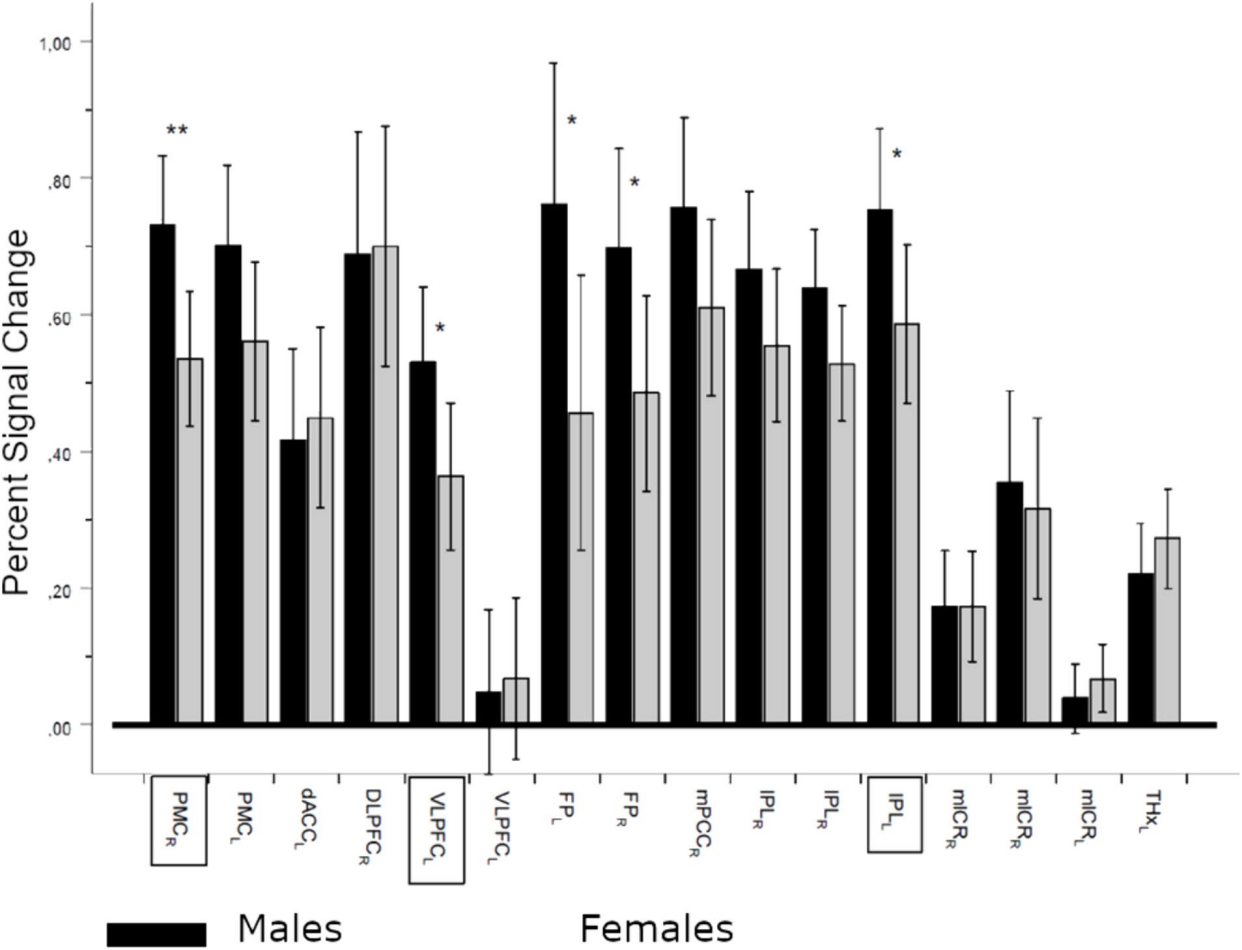
Percent signal change (PSC) differences between males and females in regions of interest (ROIs). Sex effect (between factor), regardless of the contrast: Pillai’s trace, *F*(16,46) = 1.929, *p* = 0.042, η_p_^2^ = 0.402; *denotes significance level p < 0.05 and **denotes significance level *p* < 0.01. Dark bars denote means ± CI (95%) of PSC for males, and lighter bars denote females. ROI names in frames denote regions with significant negative correlations with absolute temperature differences. PMC_rl_, bilateral premotor cortex; dACC_l_, left dorsal anterior cingulate cortex; DLPFC_r_, right dorsolateral prefrontal cortex; VLPFC_l_, left ventrolateral prefrontal cortex (frontal inferior operculum and rolandic operculum); FPl_lr_, left and right frontal poles; mPPC_r_, right medial posterior parietal cortex; IPL_lr_, left and right inferior parietal lobule (inferior parietal and anterior cingulum, mlCR_rl_, medial and lateral cerebellum; THX_r_, right thalamus).

Further comparisons of PSC between male and female ROIs demonstrated significant differences regardless of the task variant (General MANOVA, Pillai’s trace, F(16, 46) = 1.929, *p* = 0.042, η_p_^2^ = 0.402). Significantly higher activations for males were revealed in five ROIs, including three showing significant negative correlations with ATC (Figure 7).

The above results suggest that women are particularly sensitive to changes in brain temperature, due to decreased PSC in the examined ROIs. However, females maintain comparable working memory performance compared to men despite their pronounced sensitivity to even minor changes in brain temperature. We hypothesize that women compensate for temperature-dependent reductions in PSC in task-related ROIs by activating neuronal activity in other regions supporting working memory. To verify this hypothesis, we compared the whole brain activity of men and women during 2-back and 1-back task variants.

### Whole brain PSC differences between men and women performing n-back tasks

**Figure 8:**
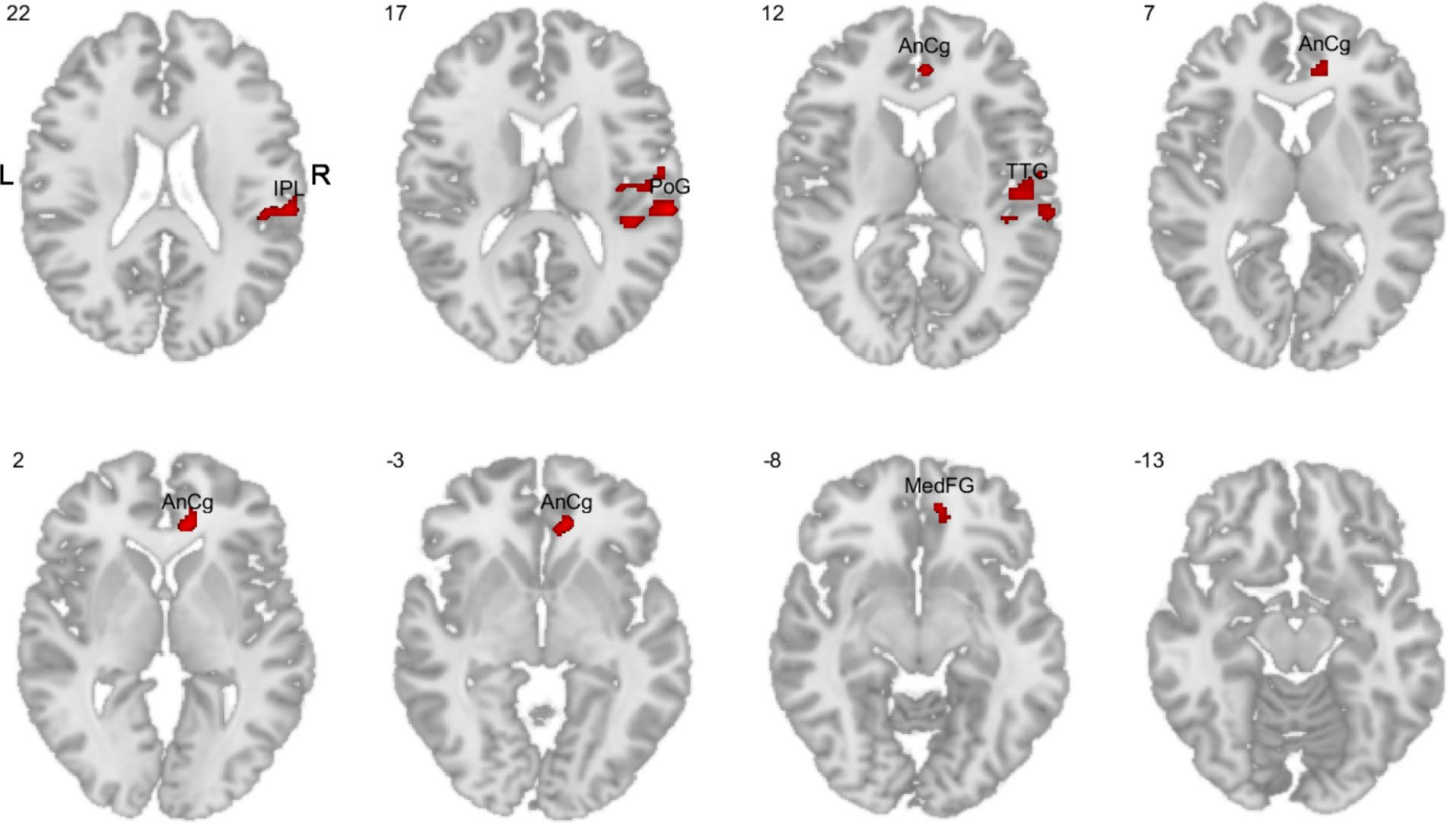
Comparisons of whole brain PSC in the 2-back task variant (1-back vs. 2-back contrast). The red color indicates higher activity in women compared to men. IPL, inferior parietal lobule; PoG, postcentral gyrus; AnCg, anterior cingulate gyrus; TTG, transverse temporal gyrus; MedFG, medial frontal gyrus.

While the comparison of men vs. women (one-sided *t*-test) whole brain PSC for the 1- vs. 0-back contrast yielded no significant results, the more demanding 2- vs. 1-back contrast showed significant differences in women compared to men. Women showed higher activity in the right inferior parietal lobule (rIPL), right postcentral gyrus (rPoG), right anterior cingulate gyrus (rAnCg), right transverse temporal gyrus (rTTG), and right medial frontal gyrus (rMedFG) (Figure 8).

Subsequent analyses did not show significant correlations between PSC and ATC in the above regions. The reversed comparison between men and women did not yield significant results in either task variant or contrast, indicating a lack of male-specific activations.

## Discussion

Our results revealed that commonly observed significant reductions in mean brain temperatures (Yablonskiy et al., 2000; Thrippleton et al., 2014; Sung et al., 2023) were predominantly attributable to male participants, while female brain temperatures remained stable. Despite statistically insignificant changes in female brain temperatures, its variation, evaluated as the ATC, was inversely associated with cognitive performance, as was neuronal activity estimated by PSC, revealing a high sensitivity of the female brain to temperature variations. This strongly suggests that ATC is a primary factor driving the positive association between cognitive performance and neuronal activity observed in females. Furthermore, our results indicate that the high sensitivity of the female brain to variations in brain temperature is compensated by the increased activations in additional neuronal regions supporting working memory.

Our study focused on brain areas identified in a meta-analysis as relevant for the execution of n-back tasks (Owen et al., 2005). Although our findings corroborated the involvement of these regions in tasks for the entire participant group, the results of correlations between PSC in selected ROIs and memory performance were predominantly driven by females. In contrast, no correlations between PSC in the selected ROIs and task performance were observed in males. Therefore, changes in brain temperature adversely affecting a woman’s neural activity, as shown in our study, could affect their behavioral performance, while similar or higher changes in men may have no performance consequences.

The lack of correlation between male PSC in task-related regions and ATC, as well as between ATC and task performance, may be due to a man’s generally higher neuronal activity, a finding supported by our results (Figure 6) and previous studies (Domes et al., 2010; Sacher et al., 2013; Dylan et al., 2021). This higher neuronal activity in men (indicated by higher PSC) could buffer the effects of temperature variations on brain function and performance. In contrast, female brain regions sensitive to ATC showed lower BOLD response, suggesting that regions with lower neuronal activity levels are more prone to temperature changes. This may also explain the absence of correlations between PSC and ATC in the less demanding 1-back tasks, which typically show an increase in PSC – a pattern observed in various tasks (Callicott et al., 1999; Beauchamp et al., 2001; Linden et al., 2003). Thus, the observed lack of correlation between ATC and PSC, both in males and in less demanding variants of the tasks in females, may be due to higher PSC mitigating the effect of ATC on neuronal activity.

However, an important finding of our study is that despite a woman’s apparent sensitivity to ATC, their working memory performance remained at the same level as men. One explanation might be that neuronal activations, like those we detected in women, support task-specific areas impacted by temperature changes. For instance, our study uniquely identified inferior parietal lobule activation in women, a region implicated in attention and inhibitory processes integral to working memory (Awh et al., 2006; Wang et al., 2016; and others). Another area exclusive to female activation, the medial frontal cortex, is pivotal for memory retention during delay periods and influences working memory capacity via interactions with the dorsolateral prefrontal cortex (Keller et al., 2015). Notably, the most pronounced PSC in women was observed in the anterior cingulate cortex (ACC). The ACC is characterized by dual functions, the rostral ACC is involved in overseeing executive cognitive control, while the ventral ACC interacts with emotional processing centers (Bush et al., 2000). During the 2-back tasks, female-specific activations in the ACC also appeared to affect its ventral regions, thereby inducing additional cognitive and emotional processes accompanying task performance. These additional emotional processes are sometimes referred to as characteristic problem-solving strategies employed by women (see Hill et al., 2016, for a review), whereas in light of our results, they are likely a side effect of compensatory activations of the ACC during the n-back tasks.

MRS-t is relatively new and is validated against invasive methods. Studies using invasive human brain temperature measurements performed on patients with acute brain injury indicate that the brain temperature ranges between 32.6°C and 45.0°C with average daily and hourly variations within one degree (Rossi et al., 2001; Lu et al., 2021; Rzechorzek et al., 2022). However, individual variations during 72-h monitoring reached as high as 4 degrees depending on the patient’s behavioral state (Rzechorzek et al., 2022). Our measurements ranged from 35.77–38.61°C with an amplitude of 0.45 degrees, which falls in the middle of the invasive results. They also fall within the range of other MRS-t measurements (Zhang et al., 2020: 35.4–37.4°C; Rzechorzek et al., 2022: 36.1–40.9°C; Sung et al., 2022: 35.5–40.1°C). Another important aspect of brain temperature studies and the application of MRS-t in particular, is reproducibility and stability of measurements. Our results of repeated measurements conducted on the group of 30 participants showed high reproducibility and stability. Also Absolute Temperature Stability index calculated for these two repeated readings indicated reproducibility within the limits of the MRS-t accuracy and showed no differences between males and females. Thus, it can be concluded that temperature measurements in our study were within the range of amplitudes for both invasive and non-invasive readings obtained in other investigations and can be considered as reliable estimates of brain temperature.

In conclusion, our results revealed that a woman’s performance in cognitive tasks was highly sensitive to variations in brain temperature. Although we observed a negative correlation between ATC and memory performance in women, their performance relative to men was on par. This was likely due to increased activation of brain areas not involved in the task in men. Overall, our study revealed subtle yet important gender-specific differences in physiological mechanisms underlying compensatory mechanisms, yielding the lack of appreciable differences between men and women subjected to cognitive tasks.

### Significance

Gender differences in cognition are of high scientific and social interest. Yet, those differences (if any) remain elusive. Here we used magnetic resonance thermometry and functional MRI to examine whether gender differences in working memory performance are determined by subtle, yet detectable between-sex differences in local brain temperature fluctuations mediated by local neuronal activity. We found that working memory performance did not differ between genders. Yet, a female’s working memory performance was more sensitive to brain temperature variation compared to males’. Furthermore, the negative impact of temperature change on female cognitive functions was compensated by higher neuronal activity in other task-specific brain areas. This compensation may account for equivocal results of studies on the between-sex differences in cognitive performance.

### Limitations

Our inferences about the neural origins of gender differences in absolute temperature change are based on the fact that ATS calculated for initial brain temperature readings from two different MRI sessions showed no differences. More direct evidence of the neural basis of these differences should include ATC calculated for initial and final temperature readings taken in the same MRI session consisting only of the resting state which was not included in our study. The second limitation of our study relates to the unknown effects of external heat induction on blood flow and neuronal activity during the elevated task (which prevented us from measuring temperature during the task). Elucidating these mechanisms could enable reliable measurements of brain temperature during the task.

## Data availability statement

The raw data supporting the conclusions of this article will be made available by the authors, without undue reservation.

## Ethics Statement

The studies involving humans were approved by ethics committee at the Nicolaus Copernicus University in Torun (approval no 3/2018, dated 6.04.2018). The studies were conducted in accordance with the local legislation and institutional requirements. The participants provided their written informed consent to participate in this study.

## Author contributions

JR: Conceptualization, Formal analysis, Methodology, Project administration, Visualization, Writing – original draft, Writing – review & editing. JD: Conceptualization, Formal analysis, Visualization, Writing – original draft. MS: Investigation, Methodology, Writing – original draft. ŁM: Formal analysis, Writing – original draft. EP-J: Methodology, Supervision, Writing – original draft. PB: Methodology, Supervision, Writing – original draft. TW: Methodology, Visualization, Writing – original draft. AW: Writing – review & editing. MK: Supervision, Writing – review & editing.
